# Visualizing the NIOSH Pocket Guide: Open-source web application for accessing and exploring the NIOSH Pocket Guide to Chemical Hazards

**DOI:** 10.1080/15459624.2023.2267098

**Published:** 2023-11-09

**Authors:** LeeAnn Lucas, Christine Whittaker, A. John Bailer

**Affiliations:** a Harvard T.H. Chan School of Public Health, Boston, Massachusetts; b Division of Science Integration, CDC/NIOSH, Cincinnati, Ohio; c Department of Statistics, Miami University, Oxford, Ohio

**Keywords:** Industrial hygiene, occupational exposure limits, occupational medicine, toxicology

## Abstract

The NIOSH Pocket Guide to Chemical Hazards is a trusted resource that displays key information for a collection of chemicals commonly encountered in the workplace. Entries contain chemical structures—occupational exposure limit information ranging from limits based on full-shift time-weighted averages to acute limits such as short-term exposure limits and immediately dangerous to life or health values, as well as a variety of other data such as chemical-physical properties and symptoms of exposure. The NIOSH Pocket Guide (NPG) is available as a printed, hardcopy book, a PDF version, an electronic database, and a downloadable application for mobile phones. All formats of the NIOSH Pocket Guide allow users to access the data for each chemical separately, however, the guide does not support data analytics or visualization across chemicals. This project reformatted existing data in the NPG to make it searchable and compatible with exploration and analysis using a web application. The resulting application allows users to investigate the relationships between occupational exposure limits, the range and distribution of occupational exposure limits, and the specialized sorting of chemicals by health endpoint or to summarize information of particular interest. These tasks would have previously required manual extraction of the data and analysis. The usability of this application was evaluated among industrial hygienists and researchers and while the existing application seems most relevant to researchers, the open-source code and data are amenable to modification by users to increase customization.

## Introduction

The NIOSH Pocket Guide to Chemical Hazards (NPG) (NIOSH 2007) is a print and electronic resource that provides concise general and hazard information useful to and trusted by industrial hygienists and other occupational safety and health professionals concerned with chemical exposures in the workplace. The NPG contains information about more than 600 chemicals and substance groupings commonly found in the work environment. It also contains NIOSH recommended exposure limits (RELs) and Occupational Safety and Health Administration (OSHA) permissible exposure limits (PELs). These values are useful for designing and evaluating the need for exposure control strategies and risk management plans (Borak and Brosseau 2015).

The NPG is used by employers, industrial hygienists, safety professionals, and others with an interest in chemical exposures in the workplace. In 2021, there were 1,341,713 page downloads of the reference guide, and the NIOSH Pocket Guide mobile application (app) (https://www.cdc.gov/niosh/npg/mobilepocketguide.html) was downloaded 85,356 times. In-depth information about the content of the Pocket Guide can be found on the guide’s introduction webpage (NIOSH 2016b).

The major strength of the NPG is having a trusted and familiar source of key information about workplace chemicals in one easy-to-use and concise format. Useful sections for each chemical entry include physical/chemical properties, NIOSH, and OSHA occupational exposure limits (OELs), measurement methods, personal protective equipment (PPE) recommendations, respirator recommendations, exposure routes, symptom information, and first-aid procedures. In addition, occupational carcinogens, and chemicals with notations for dermal hazards are identified.

Although the NPG contains a wealth of information in an electronic format, it has been presented in a static form. Thus, it has not been possible to compare chemicals individually or as a group or visualize trends in the database without manual data extraction. Also, more detailed, useful aspects of the current information categories are absent from the NPG. For example, details such as the date a REL was set would help users determine if the recommendations are consistent with other OELs and with more contemporary health effects studies (Deveau et al. 2015). Information summarizing the health basis of the recommendations would allow a more informed use of the OEL and assist in risk management and medical surveillance activation pertinent to the specific chemical hazards. The information in the NPG, while widely used, does not contain all of the chemical hazard data that may be beneficial to an industrial hygienist or researcher, such as the Globally Harmonized System of Classification and Labeling of Chemicals (GHS) codes and categories, the American Conference of Governmental Industrial Hygienists (ACGIH^®^ ) Threshold Limit Values (TLVs^®^), or the Occupational Alliance for Risk Science (OARS) Workplace Environmental Exposure Levels (WEELs), and is limited to information available from NIOSH and OSHA.

This project sought to expand the usefulness, particularly in analyses of NPG data, by developing an open-source web application that derives its data directly from the NPG database, supplementing the database with additional publicly available data from NIOSH criteria documents (NIOSH 1996, 2013), current NIOSH intelligence bulletins (NIOSH 2011), the 1988 PEL project documentation (NIOSH 1988), and the NIOSH compendium of policy documents and statements (NIOSH 1992). Existing data in the NPG was reformatted to make it searchable and compatible for analysis using a web application built with the R-Shiny statistical package (Chang et al. 2022). Additional information about each NIOSH REL was added, including the health basis and date of establishment. The intent was to provide a tool for industrial hygienists and occupational safety and health researchers to quickly visualize trends across the database, separate chemicals by carcinogen designation or health basis, and conduct other analyses of interest.

The objective of this application is to provide an open-source web-based tool that will allow analysis, visualization, and specialized sorting of the wealth of data residing in the NPG and other publicly available NIOSH documents.

## Methods

### Data source

NIOSH data were used to build this application by transferring information from the web version of the NPG to an Access database, which was then exported into an Excel spreadsheet. Columns for the NIOSH RELs, OSHA PELs, and immediately dangerous to life or health values (IDLH) fields were expanded to provide a worksheet that could be used for analyses. As part of the development of the application, the variables in Table 1 were separated into independent columns to preserve all the information in the NIOSH REL, OSHA PEL, and IDLH fields, and to separate variables for data analyses. It should be noted that while some values, standards, and health endpoint classifications have been updated, the most recent version of the NPG database includes revisions up to 2007.

Some particulate substances had two RELs or PELs—one representing total (or inhalable) particulate and the other representing respirable particulate. Since the database is divided into particulate and non-particulate substances and, for most analyses, it is desirable to have a single OEL to compare with other chemicals, the default REL and PEL used in data visualizations for particulate chemicals were the total or inhalable REL or PEL.

### Additional data

In addition to the data in the NPG Excel spreadsheet, three other pieces of information about each REL were added: the year of publication, its source, and its health basis. These variables were obtained from the 1992 NIOSH compendium of policy documents and statements (NIOSH 1992), individual NIOSH criteria documents for substances published since 1992 (NIOSH 1996, 2011, 2013), and the 1988 PEL project documentation (NIOSH 1988), which describes the health bases of changing all the RELs established in 1988 as part of that joint policy effort with OSHA.

### Quality assurance/data checking

The expanded spreadsheet was checked using double entry with two independent operators. The data sets were then compared, and discrepancies were resolved. This is a draft version of the data and is not authoritative, but instead used to understand what could be done.

### Application development

The NIOSH Pocket Guide Data Visualization Tool application was developed using R (R Core Team 2023) and RStudio (RStudio Team 2020). The Shiny package (Chang et al. 2022) was used to create the application and the R tidyverse packages (Wickham et al. 2019), particularly readxl (Wickham and Bryan 2023) were used to clean data and create graphs. The application theme was set using Shiny themes (Chang 2021) and ggthemes (Arnold 2021). Data tables were produced using the DT package (Xie et al. 2023), and plots were created using the ggplot2 package (Wickham 2016). The prototype version of this application is currently hosted on Shinyapps.io at https://johnbailer.shinyapps.io/NIOSH-Pocket-Guide/.

Early prototypes for this project were produced by Miami University students in a data visualization course. The ideas from those projects were consolidated and refined into an initial version of the application, which was tested by 14 industrial hygiene and occupational health professionals (beta-testers) representing a range of skills. Further information about the 14 professionals is in the Acknowledgments. The beta testers were asked to launch the application and respond to the questions in Table 2. They were also asked to provide feedback on the web application’s functionality, content, and any other changes they would recommend. The responses to these questions from the beta testers (not included) were used to inform and revise the application described here. For example, revisions were made in some tab names, as well as tab order and content, based on this feedback.

## Results

**Figure 1.**
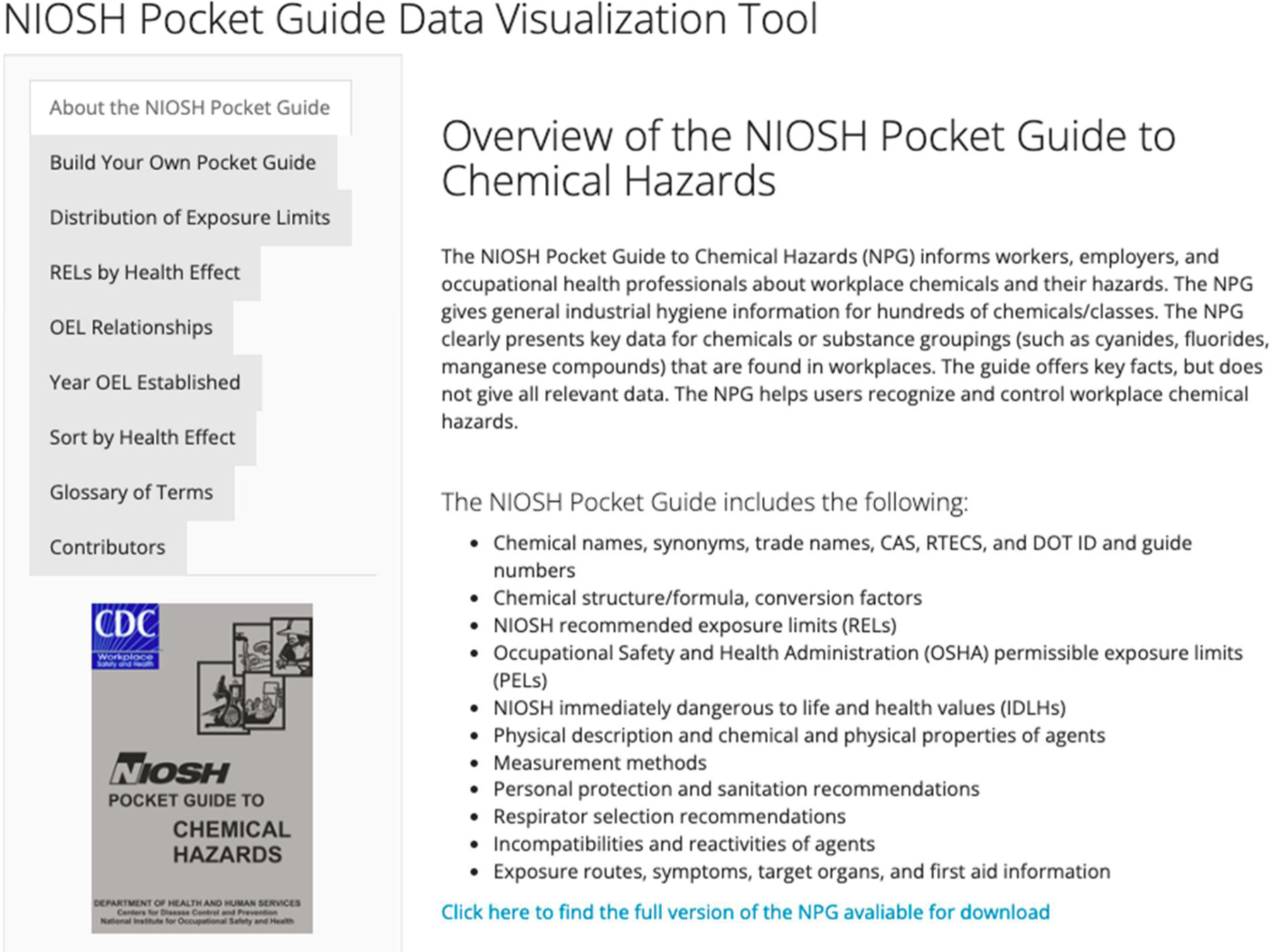
Landing page and top tab for the web application, revealing available tabs and an NPG overview.

The prototype NIOSH Pocket Guide Data Visualization Tool application (hereafter referred to as the NPG web application) (https://johnbailer.shinyapps.io/NIOSH-Pocket-Guide/) contains nine tabs that allow the user to explore different information or analyses. Upon opening the application, the “About the NIOSH Pocket Guide” tab appears on the top left (Figure 1). This tab describes the NIOSH Pocket Guide to Chemical Hazards and informs the user of the specific data values it contains. This tab also includes a picture of the hard copy of the NPG and a link to where the user can download a digital version. Throughout this discussion, we will consider how an occupational safety and health practitioner and a researcher exploring OELs might use the NPG web application.

### Build Your Own Pocket Guide

The second tab in the NPG web application is the “Build Your Own Pocket Guide” tab. Many reviewers described this tab as the most useful one for employers and occupational safety and health professionals. This tab allows a user to sort through the NPG database to create a customized version of the guide with only chemicals important to the user. A data table is produced on the page by selecting the chemicals of interest (under “Select Chemicals”), and the variables of interest (under “Columns to Show”). This table can be downloaded as an Excel or CSV file by selecting the appropriate button above the table.

**Figure 2.**
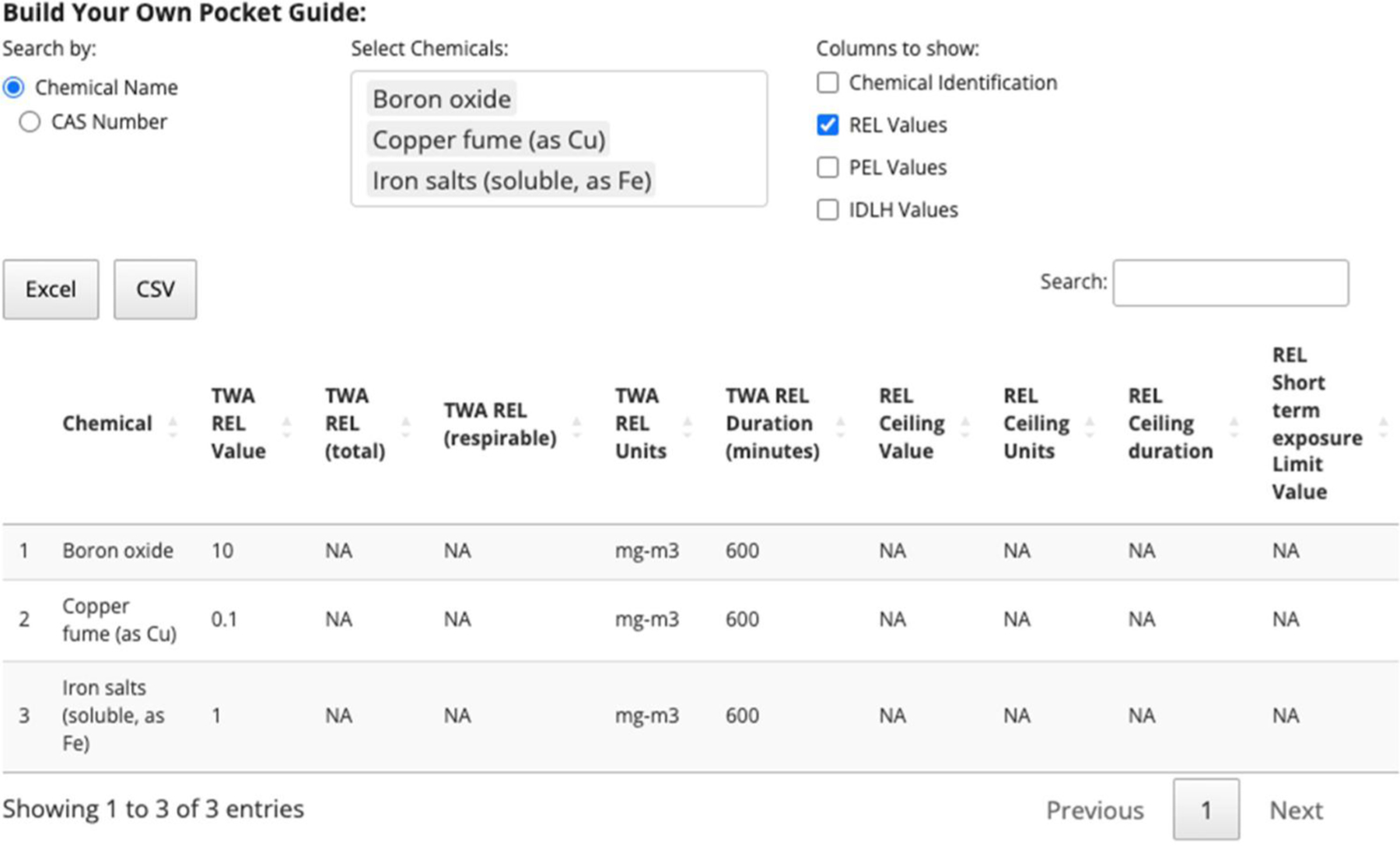
Producing a customized Pocket Guide from user-specified chemicals.

An occupational safety and health professional from a manufacturing plant, for example, may want to view the RELs of three chemicals found in their plant, boron oxide, copper fume, and iron salts. Instead of paging through the NPG or searching the electronic version, the user can select these three chemicals and export data to an Excel or CSV file that contains only the relevant information about the RELs. An example of this output is shown in Figure 2 with information about each of the three chemicals. A customized Pocket Guide makes it easier for the user to focus on their potential hazards of concern. From the downloaded Excel or CSV file, the user can print or distribute the information to other professionals or employees. In addition to filtering the data by specific chemicals, the user also has the option to select the information displayed. Using the Visualization Tool will save safety and health professionals time and effort by focusing on only the pertinent information.

### Distribution of Exposure Limits

The distribution of OELs can be explored in the “Distribution of Exposure Limits” tab. On this tab, the user selects one of four data subsets: (1) NIOSH RELs for gases and vapors; (2) NIOSH RELs for particulates; (3) OSHA PELs for gases and vapors; or (4) OSHA PELs for particulates. After selecting a subset of data, the user chooses which variable they would like to analyze. The options for visualizing the distributions are the REL or PEL, the ceiling value, the STEL, and the IDLH values. After these selections, the distribution of the selected variable is displayed in a histogram.

**Figure 3.**
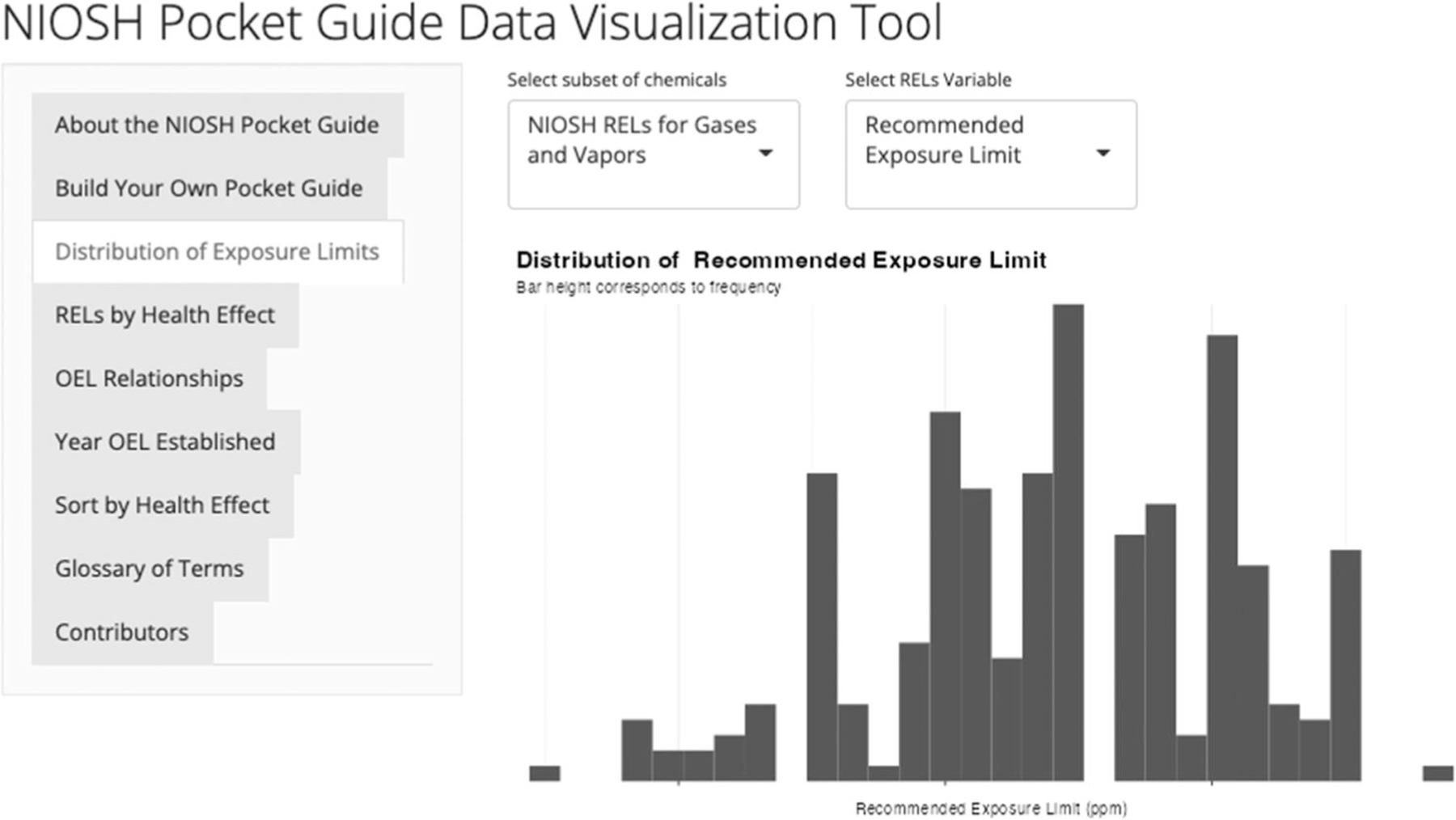
Distribution of RELs (log10 scale).

A researcher may want to compare the distribution of OELs within the NPG to other sources of chemical information. To do this, the researcher could select NIOSH RELs for gases and vapors and the recommended exposure limit, see Figure 3 for example. From this figure, it is clear that the majority of RELs in the NPG fall between 1 and 100 parts per million. Using this tab, researchers can analyze the distributions of OELs to find the typical range of OELs for gases and vapors.

### RELs by health effect

The “RELs by Health Effect” tab allows the user to investigate specific health effects. The user selects a specific health endpoint, the subset of data (gases and vapors or particulates), and then the value of interest (REL, ceiling value, or short-term exposure limit). After the variables are selected, the application creates two boxplots, one containing chemicals classified as having the health effect and the other containing chemicals that do not. This tool allows for the comparison of exposure limits based on the health effects of the chemicals. When hovering over specific data points within the graphs, the user can see which chemical corresponds to each data point, implemented using the R plotly package (Sievert 2020). It is important to note that the health effects in this database are those associated with the NIOSH REL documentation and do not necessarily reflect the health effects that might be described in the OSHA Hazard Communication standard or the Globally Harmonized System for Classification and Labeling of Chemicals (GHS).

**Figure 4.**
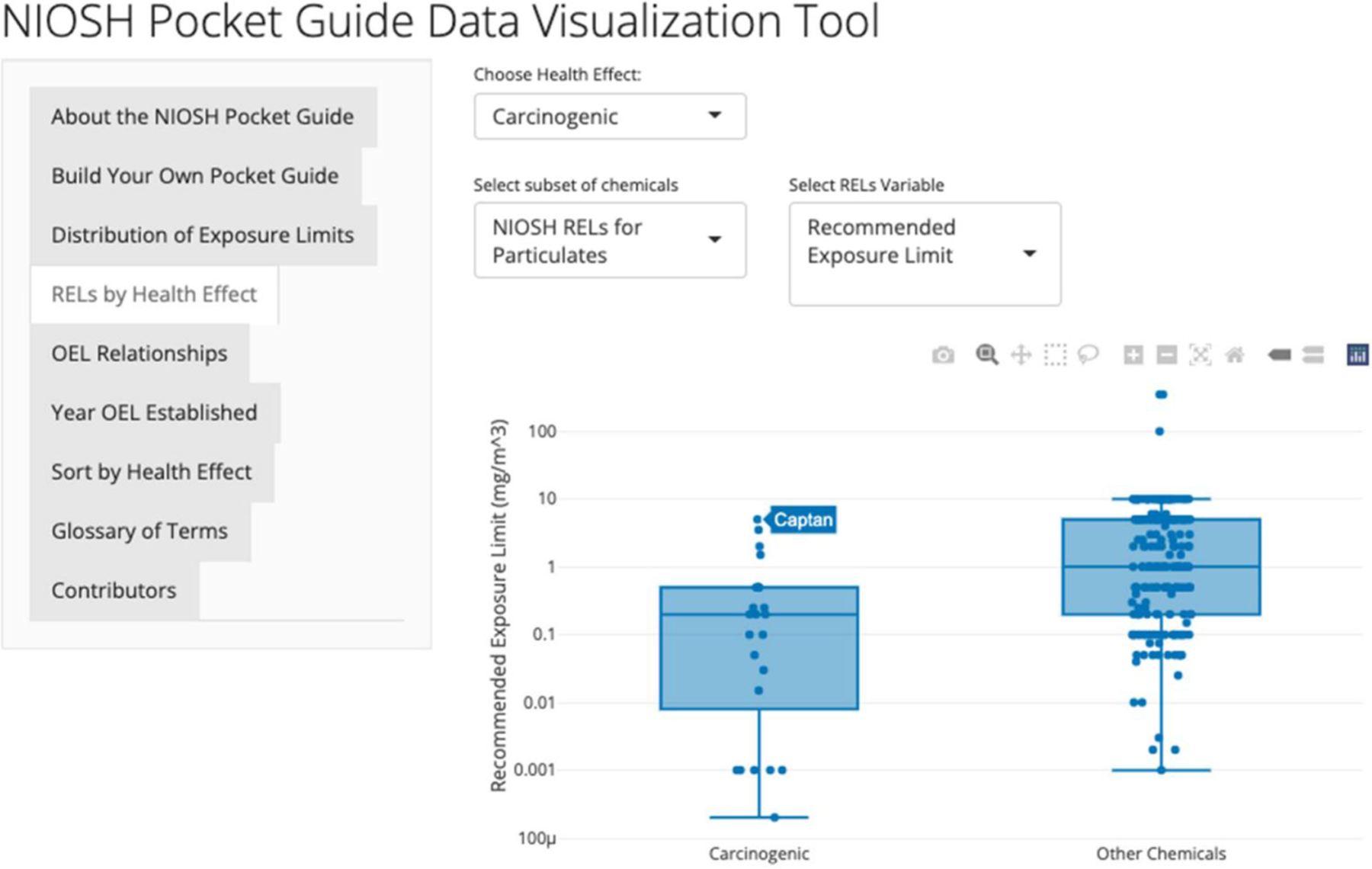
Boxplots of particulate RELs comparing carcinogens and noncarcinogens.

Figure 4 shows boxplots produced when the data selected were NIOSH RELs for particulates, the variable was the RELs, and NIOSH carcinogen designation is shown. A researcher may want to determine the identity of the outliers found in the carcinogen group. By moving the tooltip of the mouse cursor arrow to identify points, a researcher could see that captan, di-sec octyl phthalate, carbon black, and dinitrotoluene are potential outliers with high RELs that fall in the upper tail of the REL distribution. Further research questions may follow to find why these four chemicals have such high RELs compared with other carcinogenic particulates.

This tab does not contain the options to plot OSHA PELs based on health effects because the health effect bases of the OSHA PELs were not available in the data included here. The IDLH value is also not included as a graphing option based on health effect status because the IDLH reflects acute hazards and may not be directly associated with the health bases included in the database.

### OEL relationships

Scatterplots visualizing the relationships between selected OELs are produced in the “OEL Relationships” tab. After selecting the subset of chemicals, users may choose which variables they would like to compare. Options for the x-(horizontal) and y-axes (vertical) are the REL or PEL, ceiling value, STEL, and IDLH value. A scatterplot is created where a user can hover the mouse cursor over the chemical names associated with selected points. As with the illustration from Figure 4, when hovering over a point, the chemical name appears so the user can easily identify any outliers or specific data points of interest.

**Figure 5.**
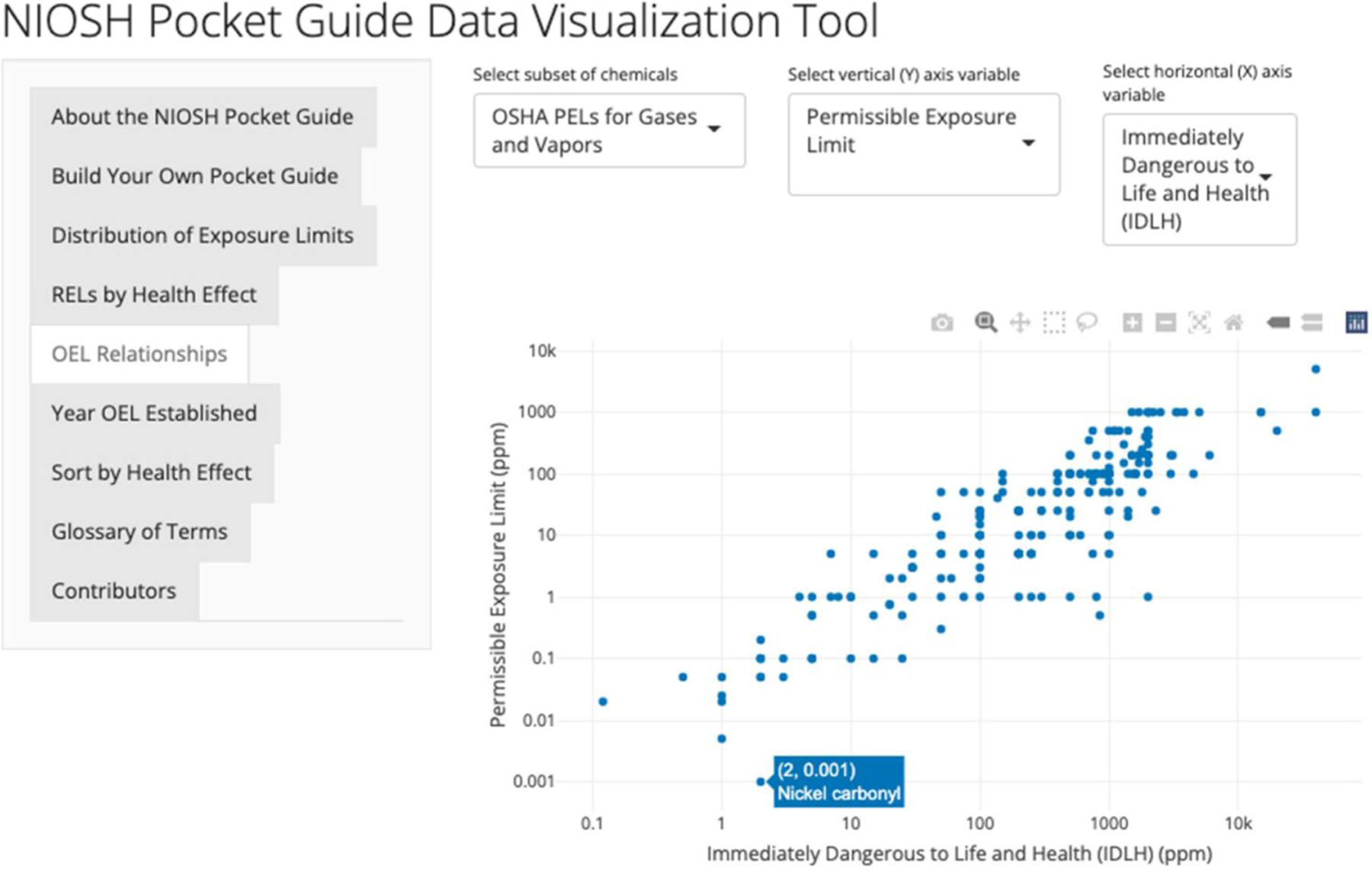
Scatterplots of PELs vs. IDLH for particulates.

One observation resulting from considering the graph of PEL values vs. IDLH values displayed in Figure 5 is that there is a strong positive, linear (on a log scale) trend where a higher PEL corresponds with a higher IDLH. This is consistent with the understanding that increasing the acute potency of a chemical generally corresponds with increasing chronic potency of the same chemical. However, there is also a good deal of variability apparent in this graph which could be explored further by comparing other variables and identifying outliers of interest.

### Analyzing Year OEL Established

**Figure 6.**
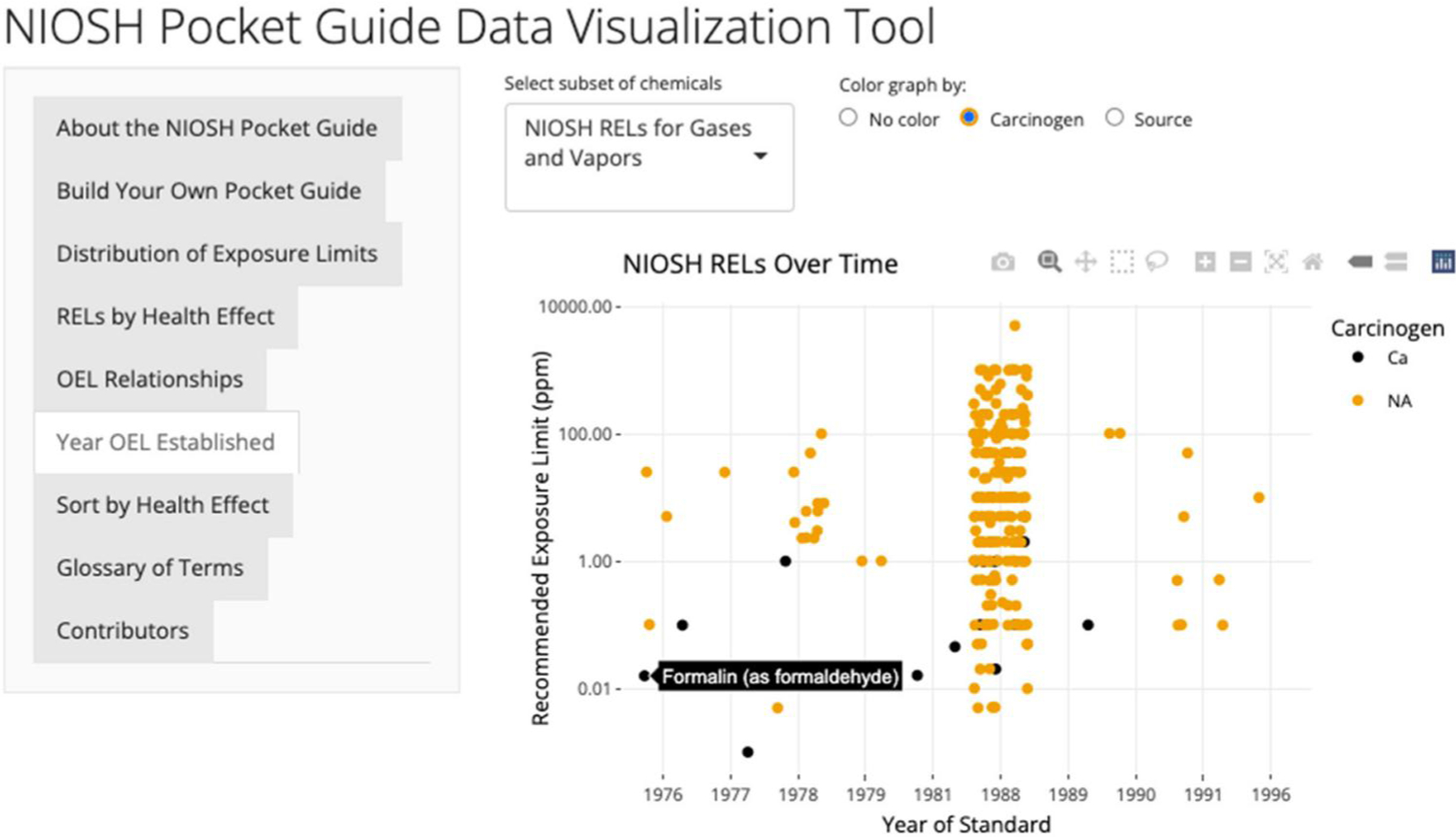
Years when RELs were established.

A timeline that summarizes when RELs were established can be found in the “Year OEL Established” tab (see Figure 6 with an illustration for gases and vapors). To customize the graph, the user can select RELs for gases and vapors or particulates. Data are not included for the PELs set by OSHA because they were established on a different timeline not included in the data available here. Readers are referred to the OSHA annotated Table Z for additional information (https://www.osha.gov/annotated-pels/table-z-1). The scatterplot displays the year the REL was set. This analysis can further be customized to color the graph based on carcinogen designation or the source of the REL. Sources for the RELs include individual NIOSH criteria documents, NIOSH current intelligence bulletins, and the NIOSH/OSHA PEL/REL update of 1988 (most NIOSH RELs were established at this time). This tab would be useful for a researcher to examine how long ago a REL was established and potentially find RELs that need to be updated.

### Sort by Health Effect

The “Sort by Health Effect” tab produces a data table that includes categories of health effects such as: carcinogenic, developmental and/or reproductive toxicity, systemic target organ toxicity (STOT), acute toxicity, genotoxicity, skin effects, skin sensitization, respiratory sensitization, and eye irritation/damage. STOT is further divided into neurological, lung, liver, cardiac, blood, and other toxicities. These designations are from the documentation supporting the NIOSH REL and do not necessarily correspond with the OSHA Hazard Communication Rule or the GHS hazard classifications. Since many of the NIOSH RELs were set years ago, this information may not align with the current understanding of the toxicity of the chemical.

The user can select a health category, and a data table is produced containing those chemicals with specific toxicity. The table includes the chemicals that are classified into the selected category and their relevant REL values. A chemical with multiple health effect designations can be found in each of the corresponding health effect tables. In addition, the user can add specific health information to the table by clicking in the “add specific health information” box.

**Figure 7.**
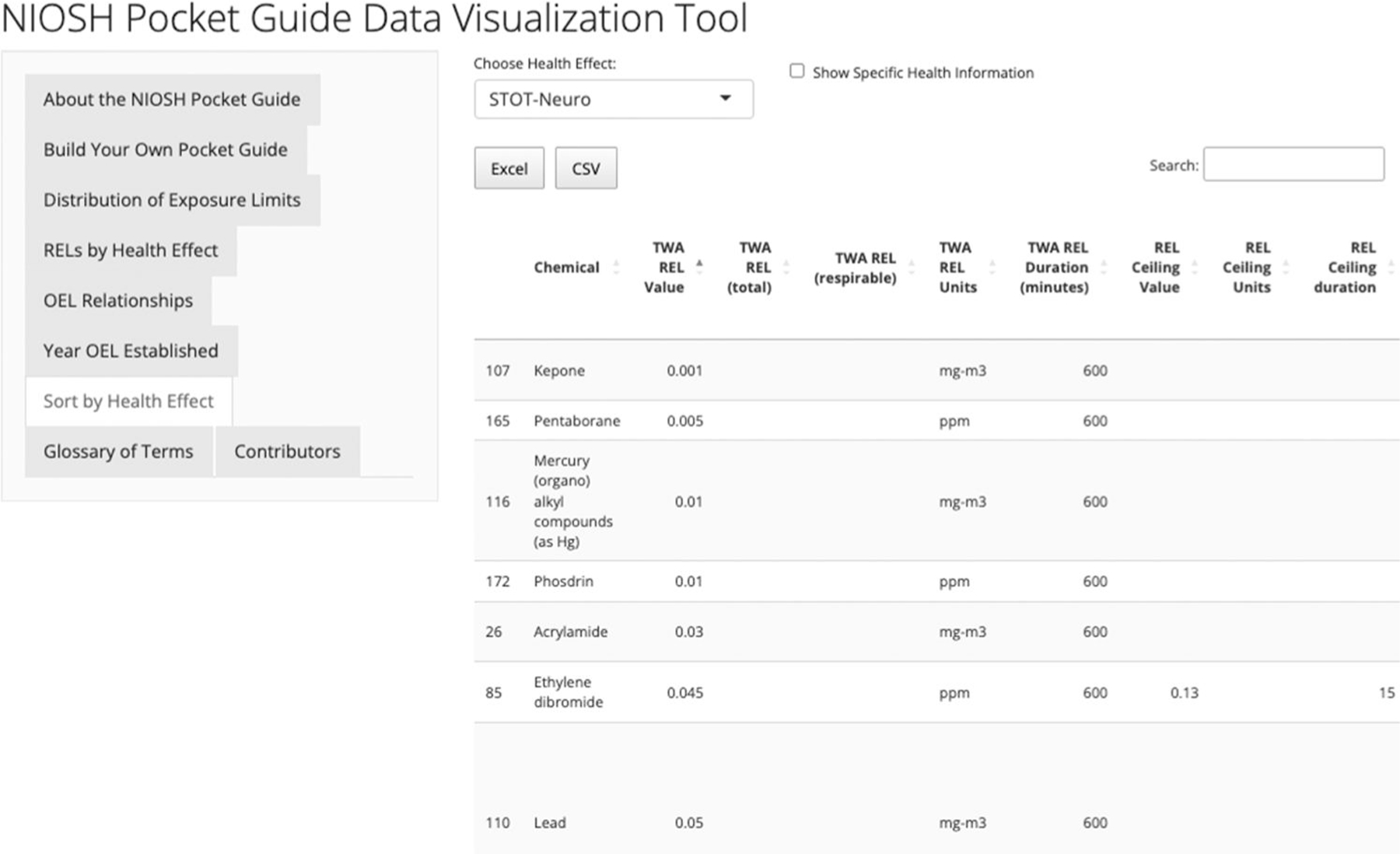
Table of OELs for chemicals associated with a neurological outcome.

For an occupational researcher who wants to find all the chemicals identified as causing neurological health effects, the procedure would be to select STOT-Neuro. Figure 7 shows the data table that would be created if STOT-Neuro was selected and the “add specific health information” was clicked. The user is then able to download the table into an Excel or CSV for further analysis. One value of this type of analysis is in searching for other chemicals that have OELs based on an endpoint of interest for a deeper review of the precedent set in interpreting related sets of health endpoint findings. This can support the rationale for new OELs and hazard assessments.

## Discussion

The prototype NIOSH Pocket Guide Data Visualization Tool was created to provide workers and researchers with a resource to quickly access information and analyze data found within the NPG. It is important to note that this Tool is not a NIOSH product but a collaboration using open-source software to demonstrate the utility of building an interface to support analyses of this database. The code and the data are available as part of the Supplemental Materials associated with this paper. There is a wealth of trusted information contained in the Pocket Guide—the OELs from both NIOSH and OSHA, occupational carcinogen designations, dermal hazard designations, and more. The NPG Visualization web application helps unpack this information and facilitates user-defined investigations of this resource.

The ability to put together a “customized Pocket Guide” focused just on chemicals of interest provides each user with access to critical information without wading through or searching a large document or database. In addition to this utility, in many cases, a comparative analysis can be done to support occupational hazard assessments. Visualizing the relationship between the REL value and the carcinogen designation demonstrates that carcinogens typically are associated with lower RELs than chemicals that have not been identified as carcinogens, however, substantial overlaps of the distributions of RELs exist. With the addition of the description of the basis for each REL and the year each was established, the database became even more useful. Sorting chemicals by health endpoint or identifying which health endpoints are important to the user in combination with the relevant OELs can aid in decisions related to substitution and risk management.

This tool is designed to be useful for occupational safety and health professionals and researchers. However, more could be done. For example, a comparison of chemicals by physical/chemical properties and health endpoint or REL may be informative. A future analysis might include a comparison of the Department of Transportation hazard codes with OELs. One of the reasons this web application code is provided along with an open source database is to allow researchers to modify the application to facilitate custom analyses of interest or to supplement these data with other information and/or to enhance features in this application.

Moving forward, the investigators will focus on including additional publicly available data and updating the source data. Additional analyses and comparisons may also be designed. One modification that has garnered some support among reviewers is to construct a “custom Pocket Guide” with only the chemicals of interest and make the visualization options in the app available for the subset of data of interest. In addition, the authors will be evaluating the use and customization of the code to determine its utility for the larger occupational safety and health community.

## Limitations

One limitation of this project includes restricting the data to freely available NIOSH sources. Much of the NIOSH chemical data included has not been updated for many years. Some of the data does not reflect current understanding of the toxicity or carcinogenicity of chemical hazards.

Another limitation was the necessary choices inherent in visualizing the data when more than one REL existed for a single chemical. This happened typically when a chemical was particulate in nature and had both a total REL and a respirable REL. When distributions were visualized, multiple entries for particulate chemicals would skew the results, so the decision was made to consistently use only the total REL to represent the chemical in the distributions. While an industrial hygienist might be most interested in the respirable REL, for purposes of the distribution analysis, consistent use of the total REL did not significantly change the shape of the distribution. In the custom Pocket Guide function, both RELs would be available to the user.

## Conclusion

The NPG has been an important reference document for 50 years. Its wealth of information has proved a useful reference for field industrial hygienists, but the uses of these data could include much more. In this application, the data in the NIOSH Pocket Guide can be accessed, compared, and visualized in ways that are useful to researchers and provides a customizable platform for users. The open-source format will allow additional innovation and further the utility of this information for occupational safety and health research.

All the data included in this application were from NIOSH sources—the NIOSH Pocket Guide database, the compendium of policy documents and statements (1992), the 1988 PEL project documentation, chemical-specific criteria documents, and current intelligence bulletins. The database used in this type of application could be customized and expanded to include additional trusted data sources. For example, comparisons with U.S. EPA IRIS reference concentrations or inhalation unit risks, EPA TSCA existing chemicals exposure limits (ECELs), the Occupational Alliance for Risk Science workplace environment exposure levels (WEELs), or other occupational or environmental exposure limits or risk estimates would be possible (Waters et al. 2015). Users could also modify the code in the app to utilize proprietary or other data of interest to them.

This application provides NIOSH data in a practical format amenable to improving understanding of the health impacts of occupational exposures. As additional information becomes available, it is hoped that this tool will increase in utility, encourage innovation, and be useful in setting priorities for the development of OELs and the exploration of new research questions in occupational safety and health.

## Supplementary Material

Suppl Material

## Figures and Tables

**Table 1.** Variable names and definitions for the expanded columns representing the NIOSH REL variable in the original spreadsheet.

Variable	Definition/Clarification
TWA REL value	10-hr Time-Weighted Average (TWA) Recommended Exposure Limit (REL) except when the compound is a particulate with two RELs (inhalable and respirable)
TWA REL value (total)	10-hr TWA REL for particulate compounds that represents the total or inhalable fraction
TWA REL value (respirable)	10-hr TWA REL for particulate compounds that represents the respirable fraction
TWA REL units	Vapors and gases are typically reported in parts per million (ppm). Particulates are typically in mg/m^3^. Fibers are in fibers/cm^3^ or million particles per cubic foot (mppcf)
TWA REL duration (minutes)	Averaging time of 600 min
REL ceiling value	Not-to-exceed concentration
REL ceiling units	ppm, mg/m^3^, or other
REL ceiling duration	Sometimes ceilings have an associated averaging time (for example, 15-min). Other times a ceiling represents an instantaneous measure
REL short-term exposure limit value	TWA limit typically averaged over 15-min or specified time
REL short-term exposure limit units	Typically in ppm or mg/m^3^
REL short-term exposure limit duration	Averaging time for the measured value
Carcinogen designation	A designation of whether a substance is identified by NIOSH as an occupational carcinogen (NIOSH 2016a)
Skin notation designation	A designation of whether a substance is identified by NIOSH as a dermal hazard (NIOSH 2017)
Comments	Any additional information contained in the NIOSH REL field

**Table 2.** Review questions for evaluating web application features and functionality.

1	What were your first impressions when you launched the app?
2	What questions might you be able to answer using this app?
3	How easy was this app to use?
4	Which functions or tabs did you find useful and/or interesting and why?
5	Which functions or tabs did you find least useful and/or interesting to you and why?
6	What is the most important feature you think we should add?
7	Did you find any of the functions confusing or hard to understand? If so, what would be helpful to make them more understandable?
8	Did you find any inconsistencies or errors?
9	Are there any other changes you would recommend?

## Data Availability

The data used in this study were derived from the NIOSH Pocket Guide to Chemical Hazards at https://www.cdc.gov/niosh/npg/default.html, the 1988 OSHA PEL Project documentation at https://www.cdc.gov/niosh/pel88/pelstart.html, the NIOSH Recommendations for occupational safety and health: compendium of policy documents and statements (1992) at https://www.cdc.gov/niosh/docs/92-100/pdfs/92-100.pdf?id=10.26616/NIOSHPUB92100, and the following NIOSH documents: NEG and NIOSH basis for an Occupational Health Standard: 2-diethylaminoethanol at https://www.cdc.gov/niosh/docs/96-104/pdfs/96-104.pdf?id=10.26616/NIOSHPUB96104; Current intelligence bulletin 64: coal mine dust exposures and associated health outcomes: a review of information published since 1995 at https://www.cdc.gov/niosh/docs/2011-172/pdfs/2011-172.pdf; and Criteria for a recommended standard: occupational exposure to hexavalent chromium at https://www.cdc.gov/niosh/docs/2013-128/pdfs/2013_128.pdf. Excel spreadsheets containing the data are available on request from the corresponding author
