# Supplementary material for: Visualizing the NIOSH Pocket Guide: Open-source web application for accessing and exploring the NIOSH Pocket Guide to Chemical Hazards
Source: J Occup Environ Hyg. Author manuscript; Available in PMC 2024 Mar 8. (PMC10922582; doi:10.1080/15459624.2023.2267098)
Supplement: Suppl Material [file NIHMS1945128-supplement-Suppl_Material.docx]

**SUPPLEMENTAL MATERIALS**

Running the NIOSH Pocket Guide Data Visualization Tool on your PC:

1. Download R Version 4.1.1
   1. Visit: <https://cran.r-project.org/mirrors.html>
   2. Select the link corresponding to the location closest to you
   3. Follow directions to download R to your computer
2. Download RStudio version 2021.09.1
   1. Visit: <https://www.rstudio.com/products/rstudio/download/#download>
   2. Select click the download link next to your operating system and follow instructions to download
3. Download NIOSH Pocket Guide Data Visualization Tool R script and supporting data files labelled “www”. Make sure that the R script and www file was loaded to the same place on your computer. Leave data files in the file called www.
4. Open RStudio and run code below to install nine packages used in the shiny application
   1. Copy code below and paste onto the R Script (top right panel in RStudio)
   2. Run code. To run, highlight all code and select Run button located on the top right of the source editor (the upper left panel in the default R settings).

install.packages(“tidyverse”)

install.packages(“ggplot2”)

install.packages(“ggthemes”)

install.packages(“readxl”)

install.packages(“DT”)

install.packages(“shiny”)

install.packages(“shinythemes”)

install.packages(“plotly”)

install.packages(“dplyr”)

1. Once all packages have been installed, open NPG_app_script.R in RStudio
   1. While in R studio choose File > Open file > select NPG_data_visualization_app.R > select Open
2. Click Run App located in the top right of the source editor
3. View and use the NIOSH Pocket Guide Data Visualization Tool
